# Point-of-Care-Testing in Acute Stroke Management: An Unmet Need Ripe for Technological Harvest

**DOI:** 10.3390/bios7030030

**Published:** 2017-08-03

**Authors:** Dorin Harpaz, Evgeni Eltzov, Raymond C. S. Seet, Robert S. Marks, Alfred I. Y. Tok

**Affiliations:** 1 Department of Biotechnology Engineering, Ben-Gurion University of the Negev, Beer-Sheva 84105, Israel; Harpaz.dorin@gmail.com; 2 School of Material Science & Engineering, Nanyang Technology University, 50 Nanyang Avenue, Singapore 639798, Singapore; 3 Institute for Sports Research (ISR), Nanyang Technology University and Loughborough University, Nanyang Avenue, Singapore 639798, Singapore; 4 Agriculture Research Organization (ARO), Volcani Centre, Rishon LeTsiyon 15159, Israel; eltzov@gmail.com; 5 Department of Medicine, Yong Loo Lin School of Medicine, National University of Singapore, NUHS Tower Block, 1E Kent Ridge Road, Singapore 119228, Singapore; raymond_seet@nuhs.edu.sg; 6 The National Institute for Biotechnology in the Negev, Ben-Gurion University of the Negev, Beer-Sheva 84105, Israel; 7 The Ilse Katz Centre for Meso and Nanoscale Science and Technology, Ben-Gurion University of the Negev, Beer-Sheva 84105, Israel

**Keywords:** stroke, diagnostics, Point-of-Care-Test, biomarkers, time-dependent treatment, multiplex and quantitative detection, data-mining

## Abstract

Stroke, the second highest leading cause of death, is caused by an abrupt interruption of blood to the brain. Supply of blood needs to be promptly restored to salvage brain tissues from irreversible neuronal death. Existing assessment of stroke patients is based largely on detailed clinical evaluation that is complemented by neuroimaging methods. However, emerging data point to the potential use of blood-derived biomarkers in aiding clinical decision-making especially in the diagnosis of ischemic stroke, triaging patients for acute reperfusion therapies, and in informing stroke mechanisms and prognosis. The demand for newer techniques to deliver individualized information on-site for incorporation into a time-sensitive work-flow has become greater. In this review, we examine the roles of a portable and easy to use point-of-care-test (POCT) in shortening the time-to-treatment, classifying stroke subtypes and improving patient’s outcome. We first examine the conventional stroke management workflow, then highlight situations where a bedside biomarker assessment might aid clinical decision-making. A novel stroke POCT approach is presented, which combines the use of quantitative and multiplex POCT platforms for the detection of specific stroke biomarkers, as well as data-mining tools to drive analytical processes. Further work is needed in the development of POCTs to fulfill an unmet need in acute stroke management.

## 1. Introduction

### 1.1. Stroke—A Leading Cause of Death

Approximately 15 million people worldwide suffer from stroke each year. More than 30% of stroke victims die every year; while over 30% suffer from permanent disability. Without intervention, the number of global deaths is projected to rise to 7.8 million in 2030 [1]. Stroke is the second most common cause of death and a major cause of disability. A common type of stroke is acute ischemic stroke (AIS) which is caused by an abrupt interruption of blood to the brain due to a thrombotic or embolic occlusion of the cerebral artery. If supply of blood is not promptly restored, this can lead to irreversible brain damage. AIS is contrasted with hemorrhagic stroke that is caused by rupture of blood vessels that are due to uncontrolled hypertension or underlying blood-vessel abnormalities such as cerebral aneurysms or arteriovenous malformations. Both the ischemic and hemorrhagic strokes can lead to permanent disabilities such as spasticity, depression, frequent falls and increased susceptibility to infections [2]. Although stroke frequently affects the elderly, young individuals are not spared by the threat of stroke [3]. In addition to traditional risk factors such as hypertension, dyslipidemia, diabetes mellitus, cigarette smoking, obesity and physical inactivity [2], individuals who suffer stroke during their youth harbor unique risk factors such as patent foramen ovale, Moya-moya disease, prothrombotic tendencies (e.g., anti-phospholipid antibodies and lupus anticoagulant) and mitochondrial disorders. Rehabilitation of stroke patients takes time–sometimes months or even years–and contributes to the rising healthcare costs in developed countries worldwide [4,5,6].

### 1.2. Gaps in the Current System

The successful management of AIS lies with its timely recognition in the pre-hospital setting (ambulance) and a prompt diagnosis at the Emergency Department (ED). In clinical practice, several features, such as the sudden onset of neurologic symptoms (e.g., facial asymmetry, arm and leg weakness and slurring of speech) in the clinical history, point to a stroke diagnosis. Ischemic stroke is differentiated from hemorrhagic stroke by neuroimaging modalities such as a computed tomography (CT) of the brain and, in some centers, using magnetic resonance imaging (MRI) to provide a more accurate assessment of infarcted brain tissues and hemorrhages [7]. In patients with an AIS, recanalization of the occluded artery confers a high likelihood of clinical recovery. To date, intravenous recombinant tissue plasminogen activator (IV-tPA, Alteplase) is the only FDA-approved treatment for AIS, provided that it is administered within 4.5 h following stroke onset [8,9,10]. More recently, randomized trial data support the use of endovascular treatment (through mechanical clot retrieval and aspiration systems) to facilitate arterial recanalization [11,12,13]. Despite these advances, there exist critical gaps in the delivery of acute stroke care, namely insufficiently accurate diagnosis of AIS and its differentiation from other stroke mimics (seizures, migraine, brain space-occupying lesions and complicated migraine), need for an appropriate selection of patients for acute reperfusion treatment, better determination of stroke etiology and finally a critical triaging of patients for resource-intensive stroke units. Several blood-based biomarkers are now available to guide such clinical decision-making in stroke management that provides clinicians with an opportunity to confirm and personalize stroke diagnosis and risk [14]. With the growing incidence of stroke, there is an emerging need for timely bedside assessment of blood-based biomarkers. As such, the demand and adoption of these new technologies are likely to increase with time. A portable and easy to use POCT is desirable to enable time-sensitive treatment to be delivered promptly, preferably during the patient’s first encounter with the paramedics or physicians. Stroke is a unique disease where treatment delays can cause significant repercussions in terms of long-term disability as a minute’s delay in delivering acute stroke treatment can result in 1.9 million loss of brain neurons.

### 1.3. Rapid Diagnosis Improves Stroke Care 

Brain cells die rapidly after stroke and any effective treatment must start as early as possible. In the clinical routine there is a “time-outcome” relationship, which continues to be the major limitation of therapeutic approaches [15,16]. The “time is brain” rational refers to the fact that brain cells die rapidly after the event of a stroke. The core of the focal ischemic lesion is irreversible and is destined to die, while the perilesional penumbra has lost its function due to reduced blood supply but maintains metabolic and structural integrity and, hence, may be salvaged. Nevertheless, there is strong evidence supporting the general association between time from onset and irreversible tissue damage—the so-called “time is brain” rationale [17,18]. Stroke has become a treatable disease: treatment with IV-tPA improves outcomes of AIS patients [12,18,19,20]. The effectiveness of most therapeutic options is time dependent and unfortunately, only a minority of eligible ischemic stroke patients receive recanalization therapies [21]. Time delays within the stroke survival chain are associated with worse outcomes [22]. Thrombolysis, IV-tPA treatment, rates remain low, with most patients being treated at the late end of the therapeutic window [17]. Current guidelines emphasize the need for early stroke care. However, significant delays occur during both the pre-hospital and in-hospital phases of care, making many patients ineligible for stroke therapies [23]. Time to thrombolysis is a critical determinant of favorable outcomes in AIS. It is a major clinical practice concern when patient outcomes are compromised due to out-of-hospital and in-hospital time delays [24]. Stroke outcome prediction mostly considers basic, most relevant, non-modifiable factors such as age and stroke severity. However, it is now well understood that stroke outcome can significantly worsen due to post-stroke complications, such as hemorrhagic transformations or post stroke infections [25]. In order to achieve the best outcome for the stroke patient, there is a need for an early diagnosis and identification of putative complications. For this on-going research, alternative-biological information, such as blood biomarkers and genetic polymorphisms, are examined in order to improve prognosis. This gap is currently addressed by barriers identification and solutions integration into the current practice. Effective reduction of time delays for AIS requires correct identification and targeted strategies [24,26,27]. The strategies to overcome those time barriers are categorized into pre-hospital, in-hospital and post-hospital strategies. Proposed pre-hospital strategies include public education on stroke symptoms awareness, prioritizing stroke by emergency medical services (EMS), increasing ease of access to medical records, pre-hospital notification and mobile CT scanning. In-hospital strategies include a streamlined code stroke system, CT scanner co-location with ED, 24/7 availability of stroke physicians, POCT or laboratory testing and access to expert neuroimaging interpretation. Post-hospital strategies include increasing availability of intravenous thrombolysis and simplification of informed consent procurement [24]. A POCT will enable a faster and better approach for stroke patients’ care in their most critical state. POCT provides the relevant needed information on-site, without sample processing, central-laboratory long testing and results analysis procedures [28]. A POCT platform is a mobile laboratory device that is located directly at the site where the patient is treated, where the needed tests are performed by the same personnel who is treating the patient, thus potentially reducing interface times and examination times. POCT holds many advantages, such as simple measurement procedures, use of low sample volumes, automated data processing and off-course mobility [29]. The potential for POCT use in stroke care is therefore enormous.

## 2. Stroke Prognostic Care Shows Painful Needs

**Figure 1:**
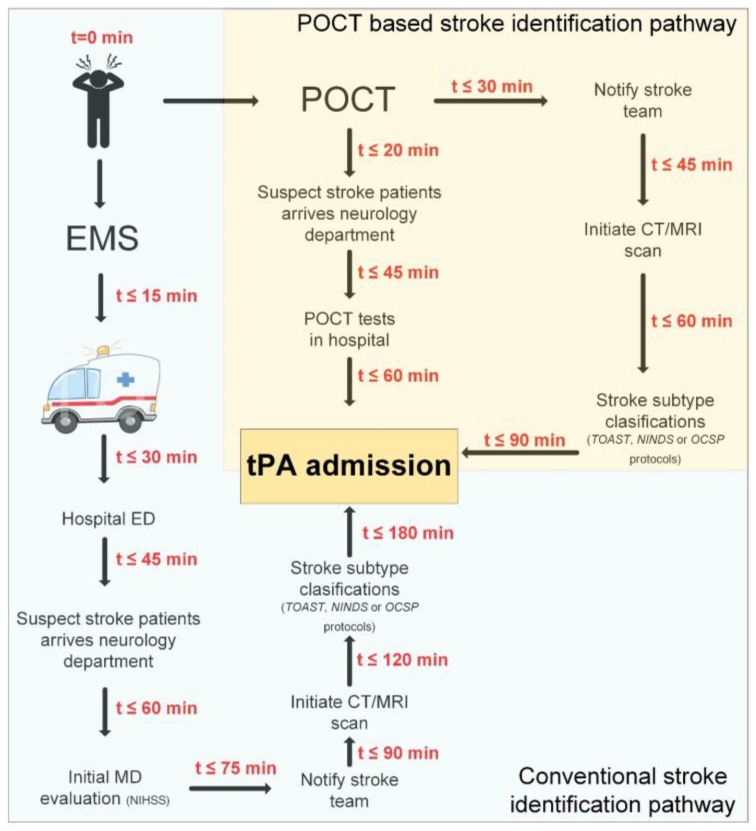
Stroke Patient Conventional Prognostic Management vs. Improved Using point-of-care-tests (POCTs). (**1**) In case of medical emergency (stroke symptoms onset), there is contact to emergency medical services (EMS) dispatch (t = 0). (**2**) EMS follows ‘case entry protocol’ and evaluate the patient medical status by specific parameters. (**3**) Ambulance patient-transportation to the hospital (t ≤ 15 min). (**4**) Conventional stroke identification pathway: in the best scenario, the patients were already confirmed to be suffering from stroke by the EMS, so he is immediately transported to a stroke specialized unit. But in case he wasn’t, the patient will go through the Emergency Department (ED), like any other patient suffering from a range of medical problems (t ≤ 30 min). The suspected stroke patient will only then be evaluated by a neurologist (t ≤ 60 min). The most common neurologic examination used is NIHSS, and followed by a CT/MRI scan for initial stroke diagnosis (t ≤ 90 min). Subsequently, stroke subtype classification (t ≤ 120 min) is conducted by either Trial of Org 10172 in Acute Stroke Treatment (TOAST), National Institute of Neurological Disorders and Stroke (NINDS) or Oxford Community Stroke Project (OCSP) classification schemes. Admission of (tissue plasminogen activator (tPA) will be on average less than 3hr from stroke-symptoms-onset (t ≤ 180 min). (**5**) POCT based stroke identification pathway: available pre-hospital POCT devices can shorten the time from stroke-symptoms-onset to neurologist examination (t ≤ 20 min). In addition, with the use of in-hospital POCT, the time to CT/MRI scan can be reduced (t ≤ 45 min) and, most importantly, the time to tPA admission can be reduced as well (60 min ≤ t ≤ 90 min).

The stroke patient conventional prognostic care consists of multiple diagnostics, classifications and treatments steps, see Table 1 and Figure 1. It can be divided into 3 main categories in stroke prognostic care: (1) Pre-hospital care; (2) In-Hospital care (imaging, clinical tests, subtypes classification and therapeutic treatment) and (3) Post-Hospital care (recovery). The full scheme includes the stroke patient management from symptoms onset and up to the recovery phase. Pre-hospital management includes symptoms identification, dispatch alert and ambulance transportation to the hospital, while In-hospital management mainly includes brain imaging, nervous system evaluation through clinical tests, and most importantly stroke subtype classification followed by lifesaving therapeutic treatment. Post-hospital management mainly includes patient physical recovery, second stroke prevention and outcome improvement. This section presents the conventional stroke patient prognostic management scheme, while also focusing on the gaps in identification for outcome improvement.

### 2.1. Pre-Hospital: From Symptoms Onset to ED Admission 

The risk of stroke is characterized by a combination of various factors, which include: previous stroke or transient ischemic attack, high blood pressure, physical inactivity, advanced age, diabetes, heart disease and smoking [2]. In most cases, stroke symptoms start suddenly (seconds to minutes), and depend on the area of the brain which is affected. If more areas of the brain are affected, more functions are likely to be lost. The major symptoms of stroke identified by the American Stroke Association include: (1) sudden weakness or numbness of arm, leg or face (especially on one side of the body); (2) trouble speaking/understanding and sudden confusion; (3) sudden vision trouble (one or both eyes); (4) sudden dizziness and loss of balance or co-ordination and (5) sudden severe headaches with no known cause [49]. Currently, the common term “Stroke chain of survival” is used to describe stroke patient diagnostic. This term refers to a rapid patient recognition and reaction to stroke warning signs. It is then followed by a rapid EMS dispatch, additional assessment [50], and rapid transportation with an urgent stroke unit/hospital pre-notification for a rapid in-hospital diagnosis and treatment [51,52,53]. Up to 70% of all stroke patients obtain first medical contact from the EMS [54]. Effective EMS systems can ultimately increase the number of patients reaching the hospital within the time window for thrombolytic therapy [55]. On the other hand, for more than 50% of all stroke cases, stroke clinical symptoms are sometimes not recognized by the EMS. The patient arrives to the hospital and is only upon arrival identified in the ED as a stroke patient. While going through a non-preprocessed patient admission, the critical survival rate for the patient significantly decreases [56]. Identification of pre-hospital stroke patients can be improved either by increasing access to EMS or by improving EMS/paramedics' patient evaluation and transport to hospital, while helping to target direct admission to the hospital as a stroke patient and thus avoid a general admission to the ED [57,58].

### 2.2. In-Hospital: Stroke Classification

Current stroke diagnosis remains largely a clinical diagnosis, with an addition of diagnostic and imaging tools. A common clinical technique conducts neurological evaluation using a scoring system, such as that using the National Institute of Health Stroke Scale (NIHSS), which is a 15-item neurologic examination stroke scale for use in acute stroke therapy [59]. In addition, an evaluation of the nervous system is conducted using a variety of imaging techniques [2], such as CT or MRI, which comprise a series of cross-sectional images of the head and brain [7]. CT/MRI scans can detect hemorrhage and are therefore useful for differentiating hemorrhagic stroke [60]. The additional existing classification technologies that may be used are: blood tests (glucose, platelet count, prothrombin time (PT), and partial thromboplastin time (PTT)), electrocardiogram, carotid ultrasound, carotid angiography, electrocardiogram, echocardiography and more [2,61]. Additional non-clinical diagnostic tools are needed to help classify stroke patients, and to better identify the patient medical status.

#### 2.2.1. Ischemic vs. Hemorrhagic Stroke

Stroke is a heterogeneous disease with more than 150 known causes. Stroke initial classification distinguishes between ischemic and hemorrhagic stroke [62]. Ischemic stroke accounts for 88% of stroke cases and occurs as a result of blood flow interference within a blood vessel which supplies blood to the brain. This usually develops due to clot formation and accumulation of fat in the vessel walls. The blood clot can be formed in the same location as the accumulation of fat, which is then termed cerebral thrombosis, or in another location in the circulatory system, which is then called cerebral embolism. In the case of cerebral embolism, the blood clot is usually formed in the heart and in large arteries of the upper chest/neck. When it breaks down, it can then be released into the bloodstream, which may cause a blockage once it reaches small vessels. A second important cause of embolism is an irregular heartbeat, known as atrial fibrillation (AF). It creates conditions where clots can form in the heart, dislodge and travel to the brain [49]. The remaining 12% of stroke cases are due to Hemorrhagic stroke, which occurs when a weakened blood vessel ruptures and bleeds into the surrounding brain [49]. As a result, the blood accumulates and compresses the surrounding brain tissue. There are two types of weakened blood vessels which usually cause Hemorrhagic stroke: aneurysms and arteriovenous malformations (AVM), with the most common cause of hemorrhagic stroke being uncontrolled hypertension (high blood pressure) [2]. An aneurysm is a ballooning of a weakened region of a blood vessel. If left untreated, the aneurysm continues to weaken until it ruptures and bleeds into the brain. An AVM is a cluster of abnormally formed blood vessels. Any one of these vessels can rupture, also causing bleeding into the brain [49]. Of the hemorrhagic strokes, 9% are due to an intra-cerebral hemorrhage (ICH), and 3% are due to a subarachnoid hemorrhage (SAH) [63]. Victims of hemorrhagic strokes are often younger and the fatality rate is higher than for ischemic stroke. Overall prognosis is also poorer for those who have hemorrhagic strokes. The symptoms of a hemorrhagic stroke usually appear suddenly and often include: very severe headaches, nausea and vomiting, partial or total loss of consciousness [2]. This distinction between hemorrhagic and ischemic stroke is thus critical for stroke management and treatment decisions. It is usually completed using various imaging technologies that may be available albeit often limited by their availability, high cost and the need for professional personnel.

#### 2.2.2. Ischemic Stroke Subtypes 

The most common stroke is AIS [49], and therefore ischemic stroke etiologies (subtypes) classification is the second most important classification in stroke prognostic clinical care [64]. The 4 main etiologies of ischemic stroke are: (1) 20% atherothrombotic (large artery atherosclerosis, LAA), (2) 20% cardioembolic (CEI), (3) 25% small vessel disease (lacunar, LAC), and (4) 5% other causes. In addition, a fifth subtype is termed ‘cryptogenic strokes’, which refers to the cases of stroke from unknown causes. ‘Cryptogenic strokes’ accounts for 30% of stroke cases [65]. The main value of subtyping ischemic stroke is in classifying patients for a better targeted therapeutic decision-making process in clinical practice, to minimize time-to-thrombosis with treatment of IV-tPA admission. The average time-to-thrombosis is 3hr from stroke onset. In addition, stroke classification serves other purposes, such as describing patients’ characteristics and grouping and phenotyping patients in a clinical study [65]. There are 3 common ischemic stroke subtypes classification schemes: (1) Trial of Org 10172 in Acute Stroke Treatment (TOAST) classification, (2) National Institute of Neurological Disorders and Stroke (NINDS) classification and (3) The Oxford Community Stroke Project (OCSP) classification, see Table 2.

### 2.3. Post-Hospital: Recovery and Prevention of Stroke Reoccurrence

After therapeutic admission and patient stabilization, the patient continues to the recovery process which usually takes place in long-term care (LTC) facilities. The recovery procedure is customized to the patient’s state and can last between a few days to up to a few months. The main focus for the stroke patient recovery is outcome improvement and second stroke prevention [73]. There are approximately 30 million stroke survivors globally and they are approximately one-quarter of the residents in LTC facilities. More than half of all global stroke survivors are left dependent on others to complete daily tasks. According to the U.S. National Stroke Association, some 10% of stroke survivors recover completely, 25% recover with minor impairments, 40% recover with moderate to severe impairments and require special care. In addition, 10% require care in a nursing home or other LTC facility, and 15% die shortly after the stroke [2]. The prediction of motor recovery assists in patient’s rehabilitation planning. Neuroimaging and neurophysiological assessments are used to measure the extent of stroke damage to the motor system and predict subsequent recovery of function. Voluntary finger extension and shoulder abduction within five days of stroke predicted subsequent recovery of upper-limb function. Diffusion-weighted imaging within seven days detected the effects of stroke on caudal motor pathways and was predictive of lasting motor impairment. Moreover fMRI activation pattern at the acute phase might also be of great interest, both in motor recovery and in language recovery. MRI DTI sequence is also promising for prediction of motor outcome [39]. As stroke patient’s recovery process focuses mainly on outcome improvement and second stroke prevention, there are many non-clinical values that can add to a better planning of the patient’s recovery.

## 3. POCTs Expedite Stroke Prognostics

**Figure 2:**
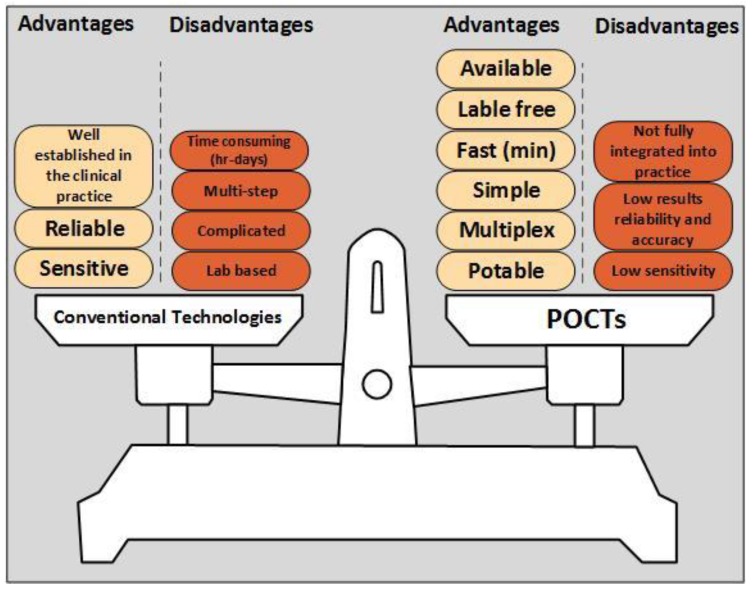
POCT vs. Conventional Technologies. The advantage of POCT vs. conventional technologies are: portability, a simple structure, easy to use, allows multiplex detection, gives results within min, and also does not require labelling. However, a POCT demonstrates lower sensitivity and gives less reliable and accurate results. Conventional technologies are usually characterized with a more sensitive and reliable detection, but the limitations deny its usage as an on-site diagnostic tool. The disadvantages of conventional technologies are: lab facility requirement, results take hours or even days and complicated usage that requires professional personnel which results in a time-consuming process.

Stroke diagnostic tools consist of a wide range of available technologies, from simple to daunting in size and complicity, see Table 1 and Figure 1. A POCT technology is defined as an on-site test. Moreover, it is usually characterized as an easy to use, robust and mobile test which targets a specific need in the clinical practice, see Figure 2. Conventional technologies are usually found in an analytical lab due to their usually more complicated set-up and operation. POCT value proposition includes mobility, affordability, selectivity, being user-friendly, robust, and allows rapid result processing. Current conventional technologies do provide sufficient sensitivity and reliable results; however, they are not always considered practical. An important factor of a POCT development is that the test will be considered effective if action is taken based on its result. For example, POCT results could reduce hospital stay, improve adherence to treatment, and reduce complications [74]. The current POCT development and design research focus on minimizing the device size, while still obtaining highly sensitive and accurate results [75]. POCT successes include the glucose biosensor strips [76] and lateral flow immunoassay strips with the most well-known being the pregnancy test. The conventional clinical technology is ELISA (enzyme-linked immunosorbent assay) [77], reaching higher sensitivities then most POCTs available, but they require multiple steps and a complicated testing procedure. It is important to note that sensitivity is not always the most critical factor, especially in cases where there needs to be faster and more accessible treatments. POCT using biosensor related technologies may result in sufficient sensitivity and accuracy standards being obtained. This section presents different POCTs tested for their use in stroke prognostic management, see Table 3.

### 3.1. POCTs in Pre-Hospital Setting: Telemedicine and Mobile Stroke Unit

Stroke is a time-dependent medical emergency in which early presentation to specialist care reduces death and improves outcome. Identifying 'true stroke' in an EMS call is challenging, with over 50% of strokes being misclassified [54]. Recent innovations have opened up new perspectives for stroke diagnosis and treatment before the patient arrives at the hospital. These include improved stroke recognition by dispatchers and paramedics, mobile telemedicine for remote clinical examination and imaging, and integration of CT scanners and POCTs in ambulances. Several clinical trials were performed in the prehospital setting, aimed at testing prehospital delivery of neuroprotective, antihypertensive, and thrombolytic therapy. These new approaches shorten time to treatment and improve outcome [17]. The use of telemedicine improves the pre-hospital diagnosis of stroke and enhances the supervision of delivery of IV-tPA in AIS. If available, integrating stroke specialists in pre-hospital stroke response teams significantly reduces time to treatment [78], however this is not possible in a large proportion of locations. Remote access to a stroke specialist is now possible, and recent studies comparing in-person consultation with remote consultation suggest that telemedicine is a promising solution. NIHSS assessment of stroke patients using telemedicine is as reliable as face-to-face assessments [79]. And radiological review of brain CT in stroke management is both feasible and reliable, with the use of, for example, the ‘ResolutionMD’ mobile application which runs on a Smartphone and affords vascular neurologists access to radiological images of patients with stroke from remote sites in the context of a telemedicine evaluation [80]. There are as yet few definitive studies that have demonstrated whether it has an effect on clinical outcomes [81,82,83,84]. The integration of mobile CT scanner and POCTs in ambulances was first clinically tested in the mobile stroke unit (MSU) project of the University of Homburg, Saarland, Germany, where, an IV-tPA treatment can be started at the scene after exclusion of intracranial hemorrhage and coagulopathies, enabling patients to be then transported to a hospital in a normal ambulance. The results of the controlled study showed a remarkable reduction of time from alarm to therapy decision (median 35 min compared to 76 min in regular care) [85]. Time to treatment in those 12 patients who received IV-tPA was approximately halved and onset-to-treatment time was only 72 min (median) [86]. However, scanning failures (mainly technical) were reported in a number of patients (12 of 53). The MSU concept was introduced in Germany, demonstrating prehospital treatment of more patients within the first hour of symptom onset [87]. Additionally, The Stroke Emergency Mobile (STEMO) project of the Charité in Berlin has added new features [88,89] such as a scanner and POCTs in a fully equipped ambulance, enabling hyperacute treatment and transport in the same vehicle. This pilot study showed encouraging results for treatment safety and number of prehospital IV-tPA applications (23 treatments within 52 days) with a mean call-to-needle time of 62 min compared to 98 min. The data suggest that prehospital stroke care in STEMO is feasible, and no safety concerns have been raised so far. A third similar clinical study uses the mobile stroke treatment unit (MSTU) in the ‘Cleveland Pre-Hospital Acute Stroke Treatment Study Group’ [90], using a registered nurse, paramedic, emergency medical technician, and a CT technologist. Then, a cerebrovascular specialist evaluates the patient via telemedicine, whereas a neuroradiologist remotely evaluates images obtained by a portable CT scanner. In addition, a variety of POCT was performed, such as coagulation profile, complete blood count, and blood chemistry.

### 3.2. POCTs in In-Hospital Setting: Coagumeters, Blood-Count, Blood-Chemistry and Biomarkers

Prior to the admission of IV-tPA, laboratory results such as coagulation profile (international normalized ratio (INR) and activated partial thromboplastin time (APTT)), blood count (platelet, leukocyte and erythrocyte count) and blood chemistry (hemoglobin, glucose, c-glutamyltransferase and p-amylase test) are required. However, in conventional clinical practices, these valuable tests are not completed due to the time consuming diagnostic procedure [13,95]. Most common prognostic guidelines of stroke recommend that thrombolytic therapy should not be delayed while waiting for these test results unless there is clinical suspicion of a bleeding abnormality or thrombocytopenia, or in cases where either the patient has received anticoagulants (heparin or warfarin) or if the use of anticoagulants is not known [13]. This might lead to an increased risk of overlooking stroke mimics or patients with contraindications for thrombolysis treatment admission [95]. In ED, 50% of non-strokes are misdiagnosed as stroke [134]. The current clinical standard is based solely on a clinician’s assessment of symptoms and rudimentary stroke scale tools. There is no simple, immediate, and unbiased way to diagnose stroke [135]. For example, studies have showed that serum glucose could be safely obtained by paramedics, and that INR POCT can reduce door-to-needle times [24,155]. Part of these tests can be completed even before arriving to the hospital when integrated to a mobile stroke unit. However, in some cases, the use of such POCT as a bed-side tool in the emergence department has enabled faster IV-tPA admission and stroke patient’s improved outcome. In a recent study [95], they compared the results obtained from POCT to those obtained from the central hospital laboratory. They showed that when using POCT instead of using the central hospital laboratory, the time-to-therapy was reduced from 84 ± 26 to 40 ± 24 min (*p* < 0.001) and the results of most laboratory tests (except APTT and INR) revealed close agreement with the results from a standard centralized hospital laboratory. However, the accuracy and effectiveness of POCT in emergency management of AIS has not been fully clinically tested [91].

#### 3.2.1. Coagumeters

By using coagumeters POCT, INR values can be measured immediately at the bedside [91]. Roche Company commercialized CoaguChek^®^, a convenient, portable and user-friendly instrument for monitoring oral anticoagulation therapy and determining the INR value from a drop of capillary whole blood. This POCT was used for both pre-hospital stroke care [90,92] and in-hospital stroke care [91,93]. CoaguChek® test principle is based on amperometric (electrochemical) detection of the coagulation response of the plateletafter activation with the human recombinant thromboplastin. The test’s user interface is a simple one with icon-based liquid crystal display (LCD). After placing a blood drop on the test strip, results are obtained within 1 min. INR POCT results correlates well with laboratory values and can be used to shorten door-to-needle time [92,94]. As mentioned previously, an APTT POCT, like the Hemochron® Junior (ITC) which was tested as an improved strategy for in-hospital stroke care, is also required [95]. Being a micro-coagulation system, it offers point-of-care monitoring of: (1) ACT-LR: Low Range Activated Clotting Time, (2) ACT+: Activated Clotting Time Plus, (3) PT, (4) Citrate PT, (5) APTT and (6) Citrate APTT. The test results are received within minutes.

#### 3.2.2. Blood-Count POCT

Blood-count tests are also needed in stroke patient prognosis. A POCT specific for blood-count is the PocH-100i hematology analyzer (Sysmex), which is a compact device designed specifically for a POCT environment. The analyzer provides a full blood count and a 3-part differential leukocyte count [96]. This POCT is used for both pre-hospital stroke care [90] and in-hospital stroke care [95]. This POCT is designed for laboratories testing of up to 25 samples per day, and detects red blood cells and platelets count. White blood cells (WBCs), red blood cells (RBCs) and platelets (PLTs) are counted using the direct current detection method with hydrodynamic focusing technology to minimize coincidence or recirculation. Hemoglobin analysis is conducted using a non-cyanide method. Hematocrit is directly determined based on the red cell count and volume detection of each individual RBC.

#### 3.2.3. Blood-Chemistry POCTs

There are two commercial blood-chemistry POCT; the i-STAT (Abbott) and the Reflotron^®^. The first one is a portable clinical analyzer designed to be used at the patient's bedside for critical care tests for blood gases, electrolytes, metabolites and coagulation, and was used for both pre-hospital stroke care [90] and in-hospital stroke care [97,98]. In a recent stroke clinical study [97], they tested a 3-tiered system together with a POCT INR in determining use of tissue-type plasminogen activator. This portable POCT uses advanced microfluidics and delivers fast, reliable, lab accurate results within 2 min. The Wide test menu includes: blood gases, electrolytes, blood chemistries, coagulation, cardiac markers (cTnI) and hematology. A clinical study was supervised by Abbott and conducted in an anonymous hospital [99], which aimed to revitalize the ED’s systems by including new emergent protocols that integrated bedside POCTs. As part of this new protocol, the i-STAT^®^ System CHEM8+ (basic metabolic panel), cTnI (troponin I), CG4+ (blood gas with lactate), and PT/INR were implemented in an effort to improve diagnostic efficiency and patient flow. Incorporating i-STAT CHEM8+, cTnI, CG4+, and PT/INR into nurse-driven emergent protocols made measurable advancements in the diagnostic-process efficiency. The ED staff was empowered to accelerate diagnosis, treatment, and disposition of patients. Another blood-chemistry POCT is the Reflotron^®^ plus analyzer (Roche, Cobas series), which is used for the measurement of c-glutamyltransferase, p-amylase, and glucose. It was tested as an improved strategy for in-hospital stroke care [95]. This is a single-test clinical chemistry system which is able to measure whole blood, plasma or serum for: liver and pancreas enzymes, metabolites and blood lipids. The full 17 parameters include: bilirubin, cholesterol, creatinine, glucose, hemoglobin, , potassium, triglycerides, uric acid, urea, alkaline phosphates, amylase, pancreatic, creatine kinase (CK), gamma glutamyl transpeptidase (GGT), glutamic oxaloacetic transaminase (GOT), aspartate aminotransferase (AST), glutamic-pyruvic transaminase (GPT) and alanine aminotransferase (ALT). The test gives on-site and reliable test results within 2–3 min.

#### 3.2.4. POCTs for Biomarkers Measurement

There are also a few POCT that are directed against a specific stroke related biomarkers measurement. A recent clinical study [109] demonstrated the usefulness of using the B-Type Natriuretic Peptide (BNP) POCT platform on suspected ischemic stroke patients in the ED in order to complete stroke subtype classification. BNP is a well-known biomarker for heart failure (HF), and its measurement is integrated into the established clinical practice of cardiologists. BNP is now identified as potentially useful for stroke patient prognostic and recovery management. BNP elevated serum levels in stroke patients show correlation with CEI stroke [87,100,101,102,103,104,105,106,107,108,112,113,114,120]. It is recommended to add a plasma BNP test at the bedside and integrate it into stroke guidelines in the ED, so that suspected stroke patients can obtain their laboratory assessments within 10 min of arrival at the ED, while the plasma BNP concentration can be measured immediately at the bedside [125]. In the case of a high plasma BNP level, emergency physicians and neurologists should strongly consider CEI stroke subtype. There are more than 20 registered devices for HF BNP measurement in the FDA, which include: Abbott AxSYM^®^ BNP, Alere Triage^®^ BNP, i-STAT BNP test and more.

Another POCT directed against stroke biomarker measurement, was developed by researchers from Cornell University, State University of New York and the New York Presbyterian Hospital. This POCT is based on enzymes tethered to nanoparticles [126] for the detection of neuron-specific enolase (NSE). Immobilization of pyruvate kinase (PK) and luciferase on silica NPs was used to achieve rapid and sensitive detection of NSE, a well-known clinically relevant biomarker for stroke. The researchers show that their data match well (r = 0.815) with the current gold standard for biomarker detection, ELISA. Moreover, they have a great advantage over ELISA as they can achieve detection in 10 min as opposed to the several hours required for traditional ELISA. Although no single biomarker will likely provide a definitive diagnosis of any disease, the glycolytic enzyme, NSE, is released from damaged neurons and has been suggested to be valuable for the diagnosis of various brain injuries. NSE has been suggested to be useful in distinguishing stroke from mimics, an important first step in expediting the diagnostic process [127,128,129]. As an alternative to antibody capture, POCTs based on fluid phase enzymatic activities [130,131] or semi-solid phase bioluminescence [132] are used for plasma NSE monitoring.

A third POCT directed against a stroke biomarker was developed by Prediction Sciences LLC (California, USA). This POCT is directed for the measurement of proteomic marker cellular fibronectin (c-Fn) [123], which has been shown in recent studies to predict hemorrhagic transformation in tPA-treated patients with a sensitivity of 100%. As mentioned previously, admission of IV-tPA is limited to a critical time window of 3 h and the amount of c-Fn in the blood of stroke patients at admission can identify if the patient is at high or low risk for a subsequent hemorrhage. This POCT platform is based on lateral flow technology detection of c-Fn, with the ability to obtain results within 10 min.

Valtari Bio™ Inc., a company from West-Virginia (USA), is also developing a POCT for the detection of stroke related biomarkers. ReST™, is a rapid evaluation stroke triage test [133], which is aimed at improving the initial stroke versus no stroke determination in ten minutes or less. This approach is based on measurement of blood brain-specific biomarkers associated with immune responses, for better stroke identification. The degree and direction of the immune system activation, following stroke and brain injury, allow the accurate identification of acute stroke from non-stroke. In addition, they employ machine learning and pattern recognition tools in order to identify different immune response patterns in the peripheral blood following various types of neuro-related brain injury. Their method is optimized due to their use of pattern recognition and ratios of biomarkers, rather than the absolute measurement of specific biomarkers. The company conducted clinical studies on over 500 real-world patients, and their preliminary clinical trial data suggests that the sensitivity and specificity for diagnosing stroke, using a pattern of expression of associated immune related biomarkers, are much higher than current clinical practice.

Furthermore, Sarissa Biomedical, a spin-off company from Coventry (UK), is developing POCT for stroke related biomarkers. SMARTChip [136] is a POCT device for stroke diagnosis that can be used by a paramedic, which will allow faster identification of stroke victims at the point of injury, and facilitate rapid coordination of the clinical treatment pathway to maximize the chances of the best possible patient outcomes. The SMARTChip can measure purines from just a drop of whole unprocessed blood and give the reading within minutes. Purines (e.g., adenosine, inosine and hypoxanthine) are neurochemicals that influence the function of the nervous system and can be used as stroke biomarkers as they are released after stroke onset. This SMARTChip POCT consist of a 3-layer structure, that converts a "dumb" microelectrode into a "smart" device capable of measuring specific analytes in real-time. The analyte sensitivity depends upon the nature of the enzymes included in the sensing layer. The upper sensing layer is termed ‘biolayer’ and contains the bioreporter (enzyme) and placed sample, with/without the target analyte. The layer underneath the ‘biolayer’ is termed ‘mediator’ layer, and the bottom layer is the electrode which transmits the signal. The current SMARTChip platforms available are for the detection of the following analytes: adenosine triphosphate (ATP), adenosine, inosine, hypoxanthine, acetylcholine, choline, glutamate, glucose, lactate and D-serine.

### 3.3. POCTs in Post-Hospital Setting: Aspirin Resistance and Biomarkers 

#### 3.3.1. POCT for Biomarkers Measurement

Recent clinical studies clearly show potential significance for adapting clinical bio markers in a stroke patient’s rehabilitation procedures, which would help to ensure appropriate individual care of the patient [39]. As mentioned previously, BNP POCT were presented in the correlation with CEI stroke subtype. However, BNP measurement can also be valuable for stroke patient’s recovery. BNP elevated serum levels in stroke patients also show a correlation with increased mortality [115,116,117,118,119,121,122,124] and indication of second stroke recurrence [110,111,156]. By monitoring BNP levels in stroke patients in their recovery process, the clinical staff will be able to better understand the stroke patient's status by predicting mortality and preventing second stroke recurrence. As previously mentioned, there are more than 20 registered devices for HF BNP measurement in the FDA.

#### 3.3.2. Aspirin Resistance POCTs

Another potentially useful POCT for stroke recovery is to screen for aspirin responsiveness after transient ischemic attack (TIA) and stroke [137]. Aspirin (ASA), the most commonly used antiplatelet agent, reduces the relative risk of major vascular events and vascular death by 20% after ischemic stroke [138]. However, the antiplatelet properties of ASA are not uniform between individuals and recurrent events, which may be caused by ‘ASA resistance’ or ASA non-responsiveness [139,140,141,142,143,144,145,146]. There is evidence that ASA nonresponsive individuals may be at increased risk of ischemic vascular events [147,148]. The response to ASA should be monitored in post-hospital care of stroke patients for the prevention of second stroke recurrence [150,153]. However, the platelet function tests for ASA monitoring are time-consuming and difficult to follow as a routine practice. There are simpler platelet function POCT, such as the PFA-100® (Platelet Function Analyzer, Dade) and the Ultegra-RPFA Verify Now Aspirin® test (RPFA) [149,151,154] available to screen stroke patients for ‘ASA resistance’. These POCT offer the possibility of a rapid and reliable identification of ASA non-responsive patients, without the requirement of a specialized laboratory [152]. Harrison et al compared the use of both the PFA-100® and the RPFA in 100 patients with transient ischemic attack or stroke receiving daily low-dose ASA treatment. Aspirin non-responsiveness is highly test-specific and large prospective studies should determine the prognostic value for each POCT used.

## 4. Key Elements in Novel Stroke-POCTs

### 4.1. What is an Ideal Brain Biomarker

Novel POCT devices should show the use of stroke related biomarkers. Ideal brain biomarkers are usually proteins which can be measured frequently from bio-fluids using safe methods, in order to provide non-clinical data on specific organs, mainly the brain and spinal cord. Those biomarkers demonstrate: (1) specificity—uniquely present in the central nervous system (CNS) and reflect the extent of brain damage; (2) sensitivity—abundant and easily detected; (3) selectivity-for example, reflect therapeutic efficacy [157]. Also, there is a need for resemblance to injury biomarker characteristics, for example, the biomarker needs to be resistant to cytoplasmic and extracellular proteolytic activity and not be dependent on renal excretion [158]. Biomarkers can be classified into three main classes: (1) susceptibility—reveal subjects with genetically mediated predisposition to a specific condition; (2) effect—measure early biological effect (structural or functional changes in affected cells or tissues); (3) exposure-measure chemicals or their metabolites to determine a patient's exposure to them [159]. The identification and use of such ideal brain biomarkers would be useful for identification of patients at risk for stroke and also to detect and monitor their treatment [158].

### 4.2. Specific Stroke Related Biomarkers

Stroke is associated with a variety of pathophysiological changes, which leads to triggering different bio-chemical processes [14]. This results in a big variety of stroke related biomarkers (Table 4) for which their clinical practice values are yet to be fully determined [160]. A convenient way to categorize stroke biomarkers is by their origin, which includes the following groups: glial cells origin, neuronal cells origin, heart muscle cells (cardiomyocytes) origin, blood vessels cells (myocytes) origin, general inflammatory cytokines, cytoskeleton proteins, hemostatic proteins, lipids, metabolic proteins and others. Glial cells’ origin stroke biomarkers include Protein S100-Beta (S100B) [157,158,161,162,163,164,165], Glial Fibrillary Acidic Protein (GFAP) [157,161,162,166,167,168,169,170,171] and Myelin Basic Protein (MBP) [172,173,174,175], whereas that for Neuronal cells include, Neuron-Specific Enolase (NSE) [129,157,158,161,162,163,166,176,177], Ubiquitin Carboxyl-terminal Hydrolase L1 (UCH-L1) [157,158,178,179,180,181] and Creatine Kinase-BB (CK-BB) [182,183]. On the other hand, Heart muscle cells (cardiomyocytes) include B-Type Natriuretic Peptide (BNP) [87,100,101,102,103,104,105,106,107,108,110,111,112,113,114,115,116,117,118,119,120,121,122,124,156,184,185,186] and Blood vessels cells (myocytes) exhibit Matrix Metallo-Proteinase 9 (MMP-9) [187,188,189,190,191]. General inflammatory cytokines and proteins stroke biomarkers include, interleukin-6 (IL-6), interleukin-1b (IL-1b), tumor necrosis factor-α (TNF-α) [166,192,193,194,195,196,197,198,199] and inflammatory protein Neutrophil Lymphocyte Ratios (NLR) [199,200,201,202,203,204,205,206], while Cytoskeleton proteins include, neurofilaments (NFs) [207,208,209], cleaved-tau (C-tau) [157,158,172,210,211,212,213], microtubule-associated protein 2 (MAP2) [214,215,216,217] and alpha-II spectrin break-down products (SBDPs) [157,218,219,220,221], Finally, Hemostatic stroke biomarkers include, D-dimer [11,222,223,224,225,226,227,228,229,230,231,232,233,234,235,236,237,238,239,240], C-reactive protein (CRP) [11,241,242,243,244,245], Fibrin monomer complex (FMC) [246], soluble fibrin (SF) [246], fibrinogen [246,247], fibrin/fibrinogen degradation products (FDPs) [246] and von willebrand factor (vWF) [247], and those of Lipid origin include, Triglycerides [248,249,250], Low density lipoprotein (LDL)/High density lipoprotein (HDL) [248,249,250], heart fatty acid binding protein (H-FABP) [251,252], free fatty acid (FFA) [253], ApoA [254,255] and ApoE4 [249,256], Whereas Metabolic proteins will have: Lactate dehydrogenase (LD) [182,257] and Albumin [258]. Other origin stroke biomarker is Decorin [259].

### 4.3. Multiplex and Quantitative Detection

The use of a multi biomarker panel strategy, instead of the measurement of a single biomarker, is probably the more useful approach. However, it is still mostly research based and of unproven value. While the results for certain biomarkers fulfil certain clinical requirements, there are currently no available biomarkers that can be recommended for immediate use in clinical settings. Multi biomarker studies will make effective panels in clinical settings [166], increasing diagnostic accuracy by minimizing the cross-effect of any individual biomarker [157]. The majority of the brain biomarkers tested so far for stroke also show association to other medical conditions. Hence, a multi-biomarker study would be especially significant [11,189]. In addition, there is a need for a quantitative biomarker detection for a useful clinical value. The use of biomarker measurement as a diagnostic value also requires a specific cut-off value which will allow an improved decision-making process in clinical practice. In order to develop such a multiplex and quantitative POCT for stroke biomarker measurement panel, there is a need to engineer novel POCT platforms.

### 4.4. POCT-based Sensors

**Figure 3:**
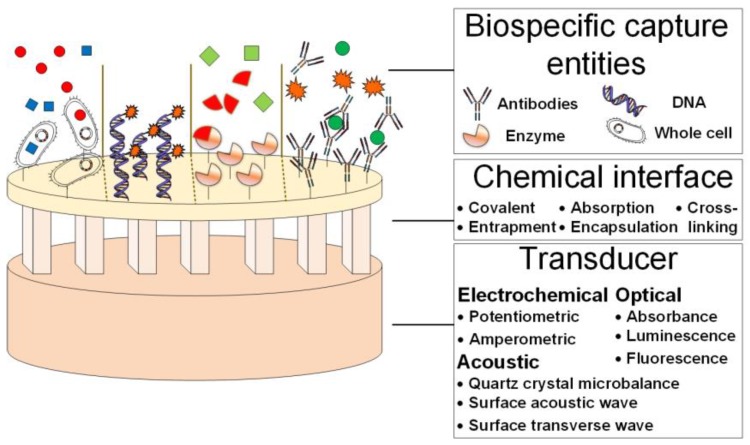
Biosensor Structure. A Biosensor consists of three components: biospecific capture entity, chemical interface and transducer. A variety of biospecific capture entities are used for the bio-detection of the target analyte in biosensors, such as antibodies, enzymes, DNA and whole cells. The biosensor chemical interface can be based on either a covalent, entrapment, absorption, encapsulation or cross-linking binding. In addition, the transducer, which is used for the signal transmission and measurement, can be based on electrochemical signal (potentiometric or amperometric), optical signal (absorbance, luminescence or fluorescence) or acoustic signal (quartz crystal microbalance, surface acoustic wave or surface transverse wave).

POCT represents an on-site tool that expedites and targets specific diagnostic delays. However, not all POCT devices demonstrate the full features required for a diagnostic device. A POCT is not defined by any particular technology or method of use, for example, it does not require reagent-free operation, battery-powered operation, or a specific degree of operator training [260]. However a biosensor as a POCT will be a more accurate approach for a novel-stroke POCT. There is a variety of POCT-based sensor analytical formats, such as microfluidics [261], microarrays [262], paper-based immunoassays [263,264,265] and optical-based sensors [266,267,268]. Biosensors represent a rapidly expanding field and have been widely used in drug discovery, diagnosis, biomedicine, food safety and processing, environmental monitoring, defense, and security applications. A biosensor is usually described as a self-containeddevice, capable of providing selective quantitative or semi-quantitative analytical information and which uses a biological recognition element and a transducer placed in intimate contact via some form of chemical immobilization [268,269,270]. A typical biosensor is based on three different parts: the biospecific capture entity-biological detection of the target molecule; the chemical interface-controls the main function of the system; and transducer – signal detection and measurement, see Figure 3. The biospecific capture entity (e.g., whole cells, enzymes, DNA or RNA strands, antibodies, antigens or biomimetic molecules) is chosen according to the target analyte, while the interfacial chemistry ensures that the biospecific capture entity molecule is immobilized upon the relevant transducer. A successful POCT sensor must possess at least three critical conditions after the immobilization steps: (1) maintaining stability and activity of the biological part during and after functionalization; (2) maximize proximity of the biological layer to the transducer; (3) maintaining sensitivity and specificity of the biological components to a target analyte [271]. Adsorption, cross-linking, covalent binding, entrapment, and less useful Langmuir–Blodgett (LMB) deposition, self-assembled monolayers, bulk modifications are the most reported methods used in biosensor functionalization applications [271,272]. The transducer, which converts the molecular recognition event to a measurable signal, may be electrochemical, acoustic or optical origin etc. [273]. An ongoing trend in the area of biosensor devices is the development of healthcare diagnostics tools. While recent advances in these technologies relate to the integration of microfluidics and optics, the miniaturization of devices and communication, and the advent of simplified fabrication technologies will allow the creation of commercial applications in the future [274].

### 4.5. Novel POCT-based Sensors Platforms 

**Figure 4:**
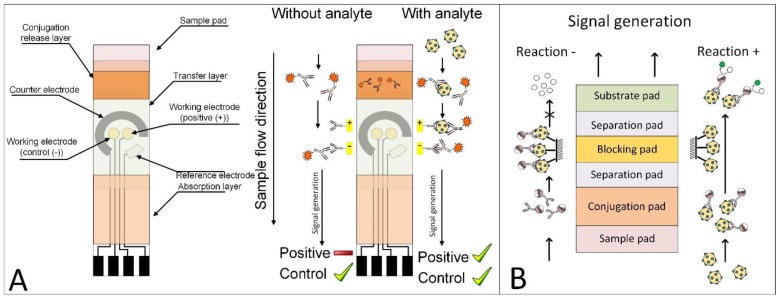
Novel POCT Platforms. (**A**) Left: Schematic illustration of electro-lateral-flow-immunoassay (ELFI) prototype. This POCT consists of a sample pad, conjugation layer immobilized with specific immune-nanobeads, a transfer layer and a SPGE immobilized with second specific Antibody for the formation of sandwich bio-recognition. Right: ELFI test concept. In case the tested sample contains the target analyte, the formulated immune-nanobeads (AuNPs-Ab-Fc) bind to it and there is a sandwich immune-complex formation on the SPGE. The signal is then measured by electrochemical reaction. In case the tested sample does not contain the target analyte, there will not be a sandwich immune-complex formation on the screen-printed gold electrode (SPGE), which will change the electrochemical signal. (**B**) Schematic illustration of Stack-Pad prototype. This POCT consists of a sample pad, conjugation pad immobilized with specific HRP-conjugated antibodies, a separation pad, blocking pad immobilized with the target analyte and a substrate pad immobilized with HRP substrate, which later produce a measurable signal. In case the tested sample contains the target analyte, the HRP-conjugated antibodies will bind to it, and continue to flow through the stack, until reaching the last membrane and producing a measurable quantitative signal. In case the tested sample doesn’t contain the target analyte, the HRP-conjugated antibodies will bind to the analyte immobilized on the blocking pad, will not continue to flow through the stack and therefore will not produce a signal.

Two novel POCT platforms that originated from the present author’s work are the electro-lateral-flow-immunoassay (ELFI) [263,275,276], which enables quantitative detection, and the stack-pad [264,265], which allows multiplex detection, see Figure 4. The ELFI platform consists of a convenient flow test strip, combined with screen-printed gold electrode (SPGE), enabling quantification and a robust set-up. The ELFI bio-recognition is based on novel immune-electroactive nanobeads that become part of a highly sensitive sandwich recognition complex. Lastly, the test results are recorded electrochemically, with a quantitative signal measurement. The electrochemical signal is contributed by a redox label present on the immobilized bead. The second novel POCT platform is the Stack-pad, which is based on stacked membranes, each layer with a specific function. The sample is added onto the bottom-most layer, and as each layer is wetted, the analyte is pushed through to the next layer of the membrane. Through the analyte migration, attached with corresponding antibody conjugated with HRP, a measurable signal is produced. In order to prevent a false positive result, a proprietary blocking layer membrane is added to stop unbounded antibodies from reaching to the top membrane. Thus, only the analyte/antibody-HRP complex will generate a signal. These two novel POCT platforms present a robust set-up which also allows multiplex and quantitative stroke biomarkers detection.

### 4.6. Use of Data-Mining for Efficient POCT Clinical Integration 

As modern research is developing, the use of big-data studies became increasingly common. Incorporating data engineering tools in scientific research helps to achieve better results’ analysis and conclusions, and has become mandatory in clinical research, with a powerful impact on the research directions and choices. Big data analytics in healthcare have evolved significantly as an innovative approach [277]. Data mining and knowledge discovery as an approach to examining medical data can limit some of the inherent bias in the hypothesis assumptions that can be found in traditional clinical data analysis [278]. Big data has four characteristics—Volume, Variety, Velocity and Value (the 4 Vs)—which traditional systems are incapable of processing. Medical data sets are continuously becoming larger, thus making it increasingly difficult for traditional systems to process them [279]. Data mining is one of the technologies called to improve the quality of service in clinical medicine through the intelligent analysis of biomedical information. From the enunciation of evidence-based medicine in early 1990s [280], the need for creating evidence that could be quickly transferred to physician daily practice is one of the most important challenges in medicine. The use of statistics to prove the validity of the treatment over discrete populations; the creation of predictive models for diagnosis, prognosis, and treatment; and the inference of clinical guidelines as decision trees or workflows from instances of healthcare protocols are examples of how data mining can help in the application of evidence based medicine. For example, in a recent study [235], N. Dougu et al developed an algorithm combining AF and D-dimer level in order to classify the diagnosis of cerebral infarction (CI). CI patients were classified into LAA, CEI, LAC infarction or others. If the D-dimer level exceeds a certain cutoff point, the patient would be suspected of having cardioembolic disease. However, despite the great penetration of these techniques in literature, their application in real clinicalpractice is far from complete [281]. In order to make use of the strong clinical potential of a POCT for stroke biomarkers measurement, there is a need to develop a multi biomarker panel, combined with a strong analytical tool, to create a customized useful POCT for improved stroke prognosis care.

## 5. Future Trends

The use of stroke prognosis POCTs is only now starting to be integrated into the established clinical practice. However, the most commonly used POCTs are mainly well-known devices for the management of the general ED's patients. However, these POCTs, such as coagulation system parameters and blood chemistry measurements, are not specifically customized to a stroke patient’s care. There are only a few innovative POCTs that are still in the research phase and have not been tested in a clinical study, and which show a direction for the development of tailored-made stroke POCT device. As stroke diagnosis and outcome improvement mostly rely on time, POCT incorporation in stroke prognosis will improve patient’s care, at their most critical state. There is a clear need for new stroke POCT devices, specifically aimed at resolving gaps in stroke clinical practice. It is within the means of current scientific knowledge to develop such POCT devices aimed at improving stroke prognosis, with the use of novel on-site immunoassays to detect stroke related biomarkers. Stroke related biomarkers research is a growing field, with new biomarkers being discovered regularly. In order to fully make use of biomarkers measurement for the integration of POCT devices in stroke care, there is a need to make use of modern analytical tools.

## Figures and Tables

**Table 1:** Stroke Conventional Prognostic Technologies.

Technology	Name	Application	Clinical Value	Reference
Imaging Technique which uses computerized x-ray imaging	Computed Tomography (CT)	3D scan of body tissues. Shows evidence of early ischemia and rules out haemorrhage. Can be performed with contrast agent for better visualization	Ischemic stroke diagnosis and admission of thrombolytic therapy	[2,30,31]
	Multi-detector Computed Tomography (MDCT)	2D array of detector elements which enables multiple slices simultaneously, and faster image acquisition. High resolution and long range scans	Ischemic stroke diagnosis and admission of thrombolytic therapy	[2]
	SPECT Computed Tomography (SPECT-CT)	This technology uses radioisotopes. Shows cross-sectional image of the target organ. The patient either swallows or is injected with a radioisotope, which travels to a target organ. The radioisotope emits radiation, which is detected. Does not reliably distinguish between hemorrhage and infarction	Determine if a specific area of the body is receiving adequate blood flow	[32]
	XENON-Contrast Computed Tomography (XENON-CT)	This technology uses the inert gas xenon to measure cerebral blood flow (CBF) in various brain regions. The patient inhales a mixture of xenon and oxygen over a period of a few minutes, allowing measurement of an increase in their density caused by their presence in the brain tissue	Determine local cerebral blood flow in small area	[32]
	Positron Emission Tomography (PET)	Measures related changes in cortical function by tracking the chemical changes which occur in tissues. Detect biochemical changes in an organ or tissue that can identify the stroke onset before anatomical changes can be seen with other imaging processes	Guide decision making for brain surgical planning	[33]
	Carotid Angiography (CA)	This technology uses dye to show the inside of human carotid arteries. A small tube (catheter) is put into an artery, usually in the groin (upper thigh), then moved upwards into one of the carotid arteries	Identification of extracranial vessel disease	[2]
Imaging Technique which uses magnetic fields and radio waves	Magnetic Resonance Imaging (MRI)	Show the slowing of water movement through the injured brain tissue, which is caused by ischemia and ruling out haemorrhage. Can detect a variety of changes in the brain and blood vessels and visualize blockages in the arteries	Ischemic stroke diagnosis and admission of thrombolytic therapy	[2,34,35,36,37]
	Magnetic Resonance Arteriogram (MRA)	Permits the visualization of blood flow in vessels and allows rapid characterization of the cervical and cephalic large vessels. Detects and grades cervical internal carotid stenosis with an accuracy of 85% to 96% compared to digital subtraction angiography	Identification of extracranial vessel disease	[34,35,38]
	Diffusion-Weighted Imaging (DWI-MRI)	Can render ischemic fields visible within minutes of ischemia onset and detects the effects of stroke on caudal motor pathways in the recovery process. Can also predict lasting motor impairment in stroke recovery	Early visualization of site and extent of ischemia	[35,38,39]
	Perfusion-Weighted Imaging (PWI-MRI)	Used to assess cerebral blood flow and blood volume in various brain regions. Usually performed by injecting a contrast agent and then obtaining a rapid series of MRIs using an ultrafast technique	Early visualization of site and extent of ischemia	[35]
	Magnetic Resonance Spectroscopy (MRS)	Spectroscopy measurement, enabling measurement of ATP, lactate levels, and pH at discrete locations within the brain. Can distinguish areas that have no viable neurons from areas that may be salvageable	Identify areas in the brain that may be salvageable	[32]
	Functional Magnetic Resonance Imaging (f-MRI)	Measurement of brain activity by detecting the changes in blood oxygenation and flow. Can measure differences in cognitive reserve	Monitoring patient recovery	[2,33,40,41,42,43]
	Magnetic Resonance Imaging Diffusion Tensor (MRI-DTI)	Provide information on white matter damage. Correlate better with cognition than conventional MRI measures	Monitoring damage progression	[40,44,45,46]
Imaging Technique which uses sound waves	Carotid Ultrasound (CU)	Show the inside of human carotid arteries and detect whether plaque has narrowed or blocked carotid arteries. Can include Doppler to show the speed and direction of blood flow through the blood vessels	Show the condition of the carotid arteries in the neck and/or intracranial vessels	[2]
	B-Mode Carotid Ultrasound (B-CU)	Provides images of various levels, or planes, enabling the creation of a three-dimensional image of the carotid artery wall and surrounding structures. Provides information on the type and extent of arterial damage, though blood clots sometimes do not appear and the method cannot distinguish a narrowed from a completely occluded artery	Show the condition of the carotid arteries wall and structure	[2]
	Duplex-Carotid Ultrasound (D-CU)	Show the human carotid arteries and detect their condition. Combines B-mode imaging and pulsed Doppler ultrasound to provide more detail on the condition of arteries	Show the condition of the carotid arteries	[47]
	Transcranial Doppler (TCD)	Probe is placed over areas on the head to detect blood velocity and pressure in certain arteries at various depths in the brain. Allows the assessment of the location and extent of occlusions or atheromatous plaques in extracranial carotid and large intracranial vessels	Show the condition of the carotid arteries and location of occlusions	[48]
	Echo-Cardiography (ECHO)	Show images of the human heart, gives information on the size, shape and function of the organ. Can also detect possible blood clots inside the heart, and problems with the aorta	Heart pathologies diagnosis	[2]
Other	Electro-Cardiogram (ECG)	Records the heart’s electrical activity, showing how fast the heart is beating, and its rhythm (steady or irregular). Can detect heart problems that may lead to a stroke and can also record the strength and timing of electrical signals which pass through the heart	Atrial fibrillation or previous heart attack diagnosis	[2]
	Blood Tests (BT)	Includes: Glucose test, Platelets count and PT/PTT. Low glucose levels can cause symptoms similar to stroke and abnormal platelet levels can be a sign of bleeding/thrombotic disorder. Can also test whether the blood is clotting normally	Bleeding/Thrombotic disorders diagnosis	[2]

**Table 2:** Ischemic Stroke Subtype Classification Methods.

Description	Stroke Subtypes	Strengths	Weaknesses	Reference
**Trial of Org 10172 in Acute Stroke Treatment (TOAST) classification**				
**Since 1993, the most clinically used method for ischemic stroke subtyping is TOAST, which is mainly based on clinical symptoms.**	LAA - Thrombosis or embolism from atherosclerosis of a large artery. CEI - Embolism from a cardiac origin. LAC - Occlusion of a small blood vessel. Other determined cause. Undetermined cause - includes either of the following cases: (1) more than one possible cause; (2) no cause is identified; (3) incomplete investigation.	Reliability has been improved by the use of a computerized algorithm	Stroke from undetermined cause is the most heterogeneous group in the TOAST system, as well as in the Stroke Data Bank. Once a patient matches more than one possible cause, he is equally grouped as a patient with a no cause identified or an incomplete investigation. This weakness could flaw the medical decision-making process.	[38,66,67,68,69]
**National Institute of Neurological Disorders and Stroke (NINDS) Classification**				
**Derived from the Harvard Stroke Registry classification.**	Brain Hemorrhages Brain Infarctions, which include atherothrombotic and tandem arterial abnormalities (LAA) CEI stroke LAC Stroke from rare causes or undetermined etiology	The best option in the search of new causes of stroke amongst patients with no known causes or with another disease not causally related to the stroke event.	Most of the currently used diagnostic tools were not available at that time, such as modern MRI with diffusion-weighted imaging, transesophageal echocardiography, TEE, magnetic resonance angiography, MRA, duplex ultrasound examination, and transcranial Doppler	[70]
**Oxford Community Stroke Project (OCSP, Bamford/Oxford) classification**				
**Relies on the initial symptoms and based on their extent.**	Total anterior circulation stroke (TAC) Partial anterior circulation stroke (PAC) LAC stroke Posterior circulation stroke (POC) The type of stroke is then coded by adding a final letter to the above: I—for infarct (e.g., TACI) H—for hemorrhage (e.g., TACH) S—for syndrome (e.g., TACS)	Patients are easily classified into groups based on clinical grounds and CT scanning, which are usually done in all stroke patients. The outcome of the stroke event is driven strongly by the severity of the stroke, which is well reflected in this classification, without addressing the cause of the stroke.	The extent and site of the brain infarct is unlikely to be specific to a particular stroke etiology. Patients classified as having a LAC infarct may have a missed cardiac source of embolism. In addition, this classification should no longer be used to investigate potential risk factors or causes of stroke.	[71,72]

**Table 3:** Stroke Prognostic POCT Devices Summary.

POCT Device	Description	Application	Clinical Value	Reference
Mobile Stroke Unit (MSU)	Imaging and a variety of Blood-tests. Integration of CT scanners and POCTs in ambulances, IV-tPA treatment can be started on-site	Consists of a registered nurse, paramedic, emergency medical technician, and a CT technologist, in addition, POCT are used, which includes coagulation profile, complete blood count, and blood chemistry	Pre-hospital: Improves stroke diagnosis and reduces time-to-IV-tPA admission	[79,86,87,88,89,90,91]
CoaguChek® (Roche)	Test strips with electro-chemical detection. Based on amperometric (electrochemical) determination of the PT time after activation of the coagulation with human recombinant thromboplastin, results are obtained within 1 min	Convenient, portable and user-friendly device for monitoring oral anticoagulation therapy which can determine the INR value from a drop of capillary whole blood	Pre/In-hospital: Improves stroke diagnosis and reduces time-to-IV-tPA admission	[91,92,93,94,95]
Hemochron® Junior (ITC)	Optical detection. Micro-coagulation system, results within minutes	POCTs monitoring of: (1) ACT-LR, (2) ACT, (3) PT, (4) Citrate PT, (5) APTT and (6) Citrate APTT	Pre/In-hospital: Improves stroke diagnosis and reduces time-to-IV-tPA admission	[96]
PocH-100i hematology analyzer (Sysmex)	Micro-fluidics. WBCs, RBCs and PLTs are counted using the direct current detection method with hydrodynamic focusing technology. Hemoglobin analysis is conducted using a non-cyanide method	Provides a full blood count and a 3-part differential leukocyte count	Pre/In-hospital: Improves stroke diagnosis and reduces time-to-IV-tPA admission	[91,96,97]
i-STAT (Abbott)	Micro-fluidics. Based on advanced microfluidic and deliver fast, reliable lab accurate results within 2 min	Bedside care tests such as blood gases, electrolytes, metabolites and coagulation	Pre/In-hospital: Improves stroke diagnosis and reduces time-to-IV-tPA admission	[91,98,99,100]
Reflotron® plus analyzer (Roche, Cobas series)	Test strips with optical detection (Reflectance photometry). Single-test clinical chemistry system which is able to measure whole blood, plasma or serum for: liver and pancreas enzymes, metabolites and blood lipids. Results within 2–3 mins	Used for blood clinical-chemistry parameters measurement, such as c-glutamyltransferase, p-amylase, and glucose	Pre/In-hospital: Improves stroke diagnosis and reduces time-to-IV-tPA admission	[96]
Abbott AxSYM® BNP/Alere Triage® BNP/i-STAT BNP	Optical Detection (AxSYM)/Fluorescent detection (Triage)/Electro-chemical sensor on a silicon chip (i-STAT). BNP POCT.	BNP elevated serum levels in stroke patients show (1) correlation with CEI stroke, (2) increased mortality and (3) indication on second stroke recurrence	In/Post-hospital: Improves stroke prognostic (correlation to CEI) and stroke recovery (indication on second stroke reoccurrence)	[88,101,102,103,104,105,106,107,108,109,110,111,112,113,114,115,116,117,118,119,120,121,122,123,124,125,126]
Cornell University, State University of New York and the New York Presbyterian Hospital	Luminescent detection. NSE POCT based on enzymes tethered to nanoparticles	NSE elevated serum levels in stroke patients assist in distinguishing stroke from mimics, an important first step in expediting the diagnostic process	In-hospital: Improves stroke diagnosis and reduces time-to-IV-tPA admission	[127,128,129,130,131,132,133]
Prediction Sciences LLC	Lateral flow POCT for the proteomic marker cellular fibronectin (c-Fn)	Fibronectin (c-Fn) elevated serum levels in stroke patients at IV-tPA admission can identify if the patient is at high or low risk for a subsequent hemorrhage	In-hospital: Reduces time-to-IV-tPA admission	[124]
ReST™ (Valtari Bio™ Inc.)	Rapid evaluation stroke triage POCT for the measurement of blood brain-specific biomarkers associated with immune responses, results within 10 min	Following stroke, the immune system is activated. The degree and direction of the immune system activation allow the accurate identification of acute stroke from non-stroke	In-hospital: Initial stroke versus no stroke diagnosis	[134,135,136]
SMARTChip (Sarissa Biomedical)	Micro-electrode POCT device for stroke diagnosis that measures purines from a drop of whole blood and give the reading within minutes	Can be used by paramedics, which will allow faster identification of stoke victims at the point of injury	In-hospital: Stroke diagnosis	[137]
PFA-100®, Platelet Function Analyzer (Dade Behring)	High-shear force dynamic flow system POCT that assesses platelet aggregation under high shear, mimicking platelet-rich thrombus formation after injury to a small vessel wall under flow conditions	Rapid and reliable identification of aspirin non-responsive patients, without the requirement of a specialized laboratory	Post-hospital: Prevention of second stroke recurrence	[138,139,140,141,142,143,144,145,146,147,148,149,150,151,152,153,154,155]
Ultegra-RPFA VerifyNow Aspirin® test (Accumetrics)	Optical detection. POCT based on turbidimetric optical detection of platelet aggregation in whole blood. As aggregation occurs, the system converts luminosity transmittance results into ‘Aspirin Reaction Units’	Rapid and reliable identification of aspirin non-responsive patients, without the requirement of a specialized laboratory	Post-hospital: Prevention of second stroke recurrence	[138,139,140,141,142,143,144,145,146,147,148,149,150,151,152,153,154]

**Table 4:** Stroke Related Biomarkers Summary.

Family	Biomarker	Expression Association	Stroke Clinical Value	Reference
***Glial cells origin***	S100-Beta	Associated with blood–brain barrier (BBB) dysfunction	Diagnosis	[157,158,161,162,163,164,165]
	Glial Fibrillary Acidic Protein (GFAP)	Associated with the size of brain lesions, the neurological status and short-term functional outcome	Prognosis: outcome prediction	[157,161,162,166,167,168,169,170,171]
	Myelin Basic Protein (MBP)	Associated with worsened outcome	Prognosis: outcome prediction	[172,173,174,175]
***Neuronal cells origin***	Neuron-Specific Enolase (NSE)	Associated with neurological outcomes and infarct volume	Prognosis: outcome prediction	[129,157,158,161,162,163,166,176,177]
	Ubiquitin Carboxyl-terminal Hydrolase L1 (UCH-L1)	Associated with the extent of the neuronal injury	Prognosis: outcome prediction	[157,158,178,179,180,181]
	Creatinine Kinase-BB (CKBB)	Associated with the extent and severity of the brain damage and the recovery potential	Prognosis and recovery	[182,183]
***Heart muscle cells (cardio-myocytes) origin***	B-Type Natriuretic Peptide (BNP)	Associated with CEI ischemic stroke, increased mortality and second stroke indication	Prognosis: subtype classification and recovery: second stroke prevention	[87,100,101,102,103,104,105,106,107,108,110,111,112,113,114,115,116,117,118,119,120,121,122,124,156,184,185,186]
***Blood vessels cells (myocytes) origin***	Matrix Metallo-Proteinase 9 (MMP-9)	Associated with blood–brain barrier (BBB) dysfunction and second stroke indication	Diagnosis and recovery: second stroke prevention	[187,188,189,190,191]
***General inflammatory cytokines and proteins***	Interleukin-6 (IL-6), interleukin-1b (IL-1b), tumor necrosis factor-α (TNF-α)	Associated with CEI ischemic stroke subtype	Prognosis: subtype classification	[166,192,193,194,195,196,197,198,199]
	Neutrophil Lymphocyte Ratios (NLR)	Associated with increased mortality and LAA ischemic stroke subtype	Prognosis: subtype classification and recovery	[199,200,201,202,203,204,205,206]
***Cytoskeleton proteins***	Neurofilaments (NF)	Associated with disconnected axons	Prognosis	[207,208,209]
	Cleaved-tau (C-tau)	Associated with neuronal degeneration and disease progression	Prognosis	[157,158,172,210,211,212,213]
	Microtubule-associated protein 2 (MAP2)	Associated with dendritic injury	Prognosis	[214,215,216,217]
	Alpha-II spectrin break-down products (SBDPs)	Associated with apoptosis and necrotic neuronal death	Prognosis	[157,218,219,220,221]
***Hemostatic proteins***	D-dimer (DD)	Associated with CEI ischemic stroke subtype and recurrence strokes	Prognosis: subtype classification	[11,222,223,224,225,226,227,228,229,230,231,232,233,234,235,236,237,238,239,240]
	C-reactive protein (CRP)	Associated with AIS diagnosis, stroke severity and LAA ischemic stroke subtype	Prognosis: subtype classification	[11,241,242,243,244,245]
	Fibrin monomer complex (FMC)	Associated with stroke early recognition and CEI ischemic stroke subtype	Diagnosis and prognosis: subtype classification	[246]
	Soluble fibrin (SF)	Associated with stroke early recognition and CEI ischemic stroke subtype	Diagnosis and prognosis: subtype classification	[246]
	Fibrinogen	Associated with stroke early recognition	Diagnosis	[246,247]
	Fibrin/fibrinogen degradation products (FDPs)	Associated with stroke early recognition	Diagnosis	[246]
	Von willebrand factor (vWF)	Associated with stroke early recognition and LAC ischemic stroke subtype	Diagnosis and prognosis: subtype classification	[247]
***Lipids origin***	Triglycerides	Associated with LAA ischemic stroke subtype	Prognosis: subtype classification	[248,249,250]
	Low density lipoprotein (LDL)/High density lipoprotein (HDL)	Associated with LAA ischemic stroke subtype	Prognosis: subtype classification	[248,249,250]
	Heart fatty acid binding protein (H-FABP)	Associated with early diagnosis of stroke	Diagnosis	[251,252]
	Free fatty acid (FFA)	Associated with CEI ischemic stroke subtype	Prognosis: subtype classification	[253]
	ApoA	Associated with LAA ischemic stroke subtype, and stroke severity	Prognosis: subtype classification and outcome prediction	[254,255]
	ApoE4	Associated with ischemic stroke diagnosis (vs. hemorrhagic stroke) and with LAC and LAA ischemic stroke subtypes	Diagnosis and prognosis: subtype classification	[249,256]
***Metabolic proteins***	lactate dehydrogenase (LD)	Associated with the extent and severity of the brain damage and the recovery potential	Prognosis and recovery	[182,257]
	Albumin	Associated with CEI ischemic stroke subtype	Prognosis: subtype classification	[258]
***Others***	Decorin	Associated with LAA ischemic stroke subtype	Prognosis: subtype classification	[259]
